# Rapid Implementation and Innovative Applications of a Virtual Intensive Care Unit During the COVID-19 Pandemic: Case Study

**DOI:** 10.2196/20143

**Published:** 2020-09-03

**Authors:** Atiya Dhala, Farzan Sasangohar, Bita Kash, Nima Ahmadi, Faisal Masud

**Affiliations:** 1 Department of Surgery Houston Methodist Hospital Houston, TX United States; 2 Department of Industrial and Systems Engineering Texas A&M University College Station, TX United States; 3 Center for Outcomes Research Houston Methodist Hospital Houston, TX United States; 4 School of Public Health Texas A&M University College Station, TX United States; 5 Departments of Anesthesiology and Critical Care Houston Methodist DeBakey Heart and Vascular Center Houston Methodist Hospital Houston, TX United States

**Keywords:** intensive care units, critical care, pandemics, SARS-CoV-2, telemedicine, infection control, COVID-19

## Abstract

**Background:**

The COVID-19 pandemic has necessitated a rapid increase of space in highly infectious disease intensive care units (ICUs). At Houston Methodist Hospital (HMH), a virtual intensive care unit (vICU) was used amid the COVID-19 outbreak.

**Objective:**

The aim of this paper was to detail the novel adaptations and rapid expansion of the vICU that were applied to achieve patient-centric solutions while protecting staff and patients’ families during the pandemic.

**Methods:**

The planned vICU implementation was redirected to meet the emerging needs of conversion of COVID-19 ICUs, including alterations to staged rollout timing, virtual and in-person staffing, and scope of application. With the majority of the hospital critical care physician workforce redirected to rapidly expanded COVID-19 ICUs, the non–COVID-19 ICUs were managed by cardiovascular surgeons, cardiologists, neurosurgeons, and acute care surgeons. HMH expanded the vICU program to fill the newly depleted critical care expertise in the non–COVID-19 units to provide urgent, emergent, and code blue support to all ICUs.

**Results:**

Virtual family visitation via the Consultant Bridge application, palliative care delivery, and specialist consultation for patients with COVID-19 exemplify the successful adaptation of the vICU implementation. Patients with COVID-19, who were isolated and separated from their families to prevent the spread of infection, were able to virtually see and hear their loved ones, which bolstered the mental and emotional status of those patients. Many families expressed gratitude for the ability to see and speak with their loved ones. The vICU also protected medical staff and specialists assigned to COVID-19 units, reducing exposure and conserving personal protective equipment.

**Conclusions:**

Telecritical care has been established as an advantageous mechanism for the delivery of critical care expertise during the expedited rollout of the vICU at Houston Methodist Hospital. Overall responses from patients, families, and physicians are in favor of continued vICU care; however, further research is required to examine the impact of innovative applications of telecritical care in the treatment of critically ill patients.

## Introduction

**Figure 1:**
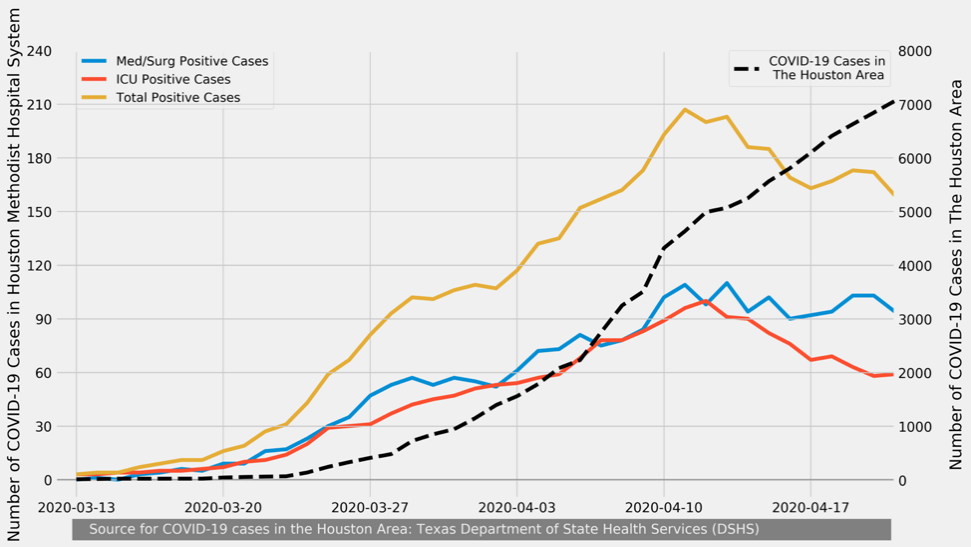
Number of Houston area COVID-19 cases (dashed line) from March 13-April 21, 2020 compared to the numbers of Houston Methodist cases (solid lines). ICU: intensive care unit; Med/Surg: Medical/Surgical.

The outbreak of the SARS-CoV-2 pandemic has been widely reported in news articles and journals [1]. Although the initial flow of patients with COVID-19 in Houston, Texas, began slowly compared to that in other large metropolises such as New York City, Houston Methodist Hospital (HMH) began to marshal all its resources to plan for the exponential growth of patients who tested positive for or were suspected of having COVID-19. HMH coordinated responses with other hospitals in the Texas Medical Center (TMC), the largest medical district in the world. As the number of COVID-19 cases increased across the region, the intensive care unit (ICU) beds dedicated to COVID-19 patients in HMH also increased (Figure 1). At the peak of the pandemic, HMH had dedicated 150 ICU beds to patients with COVID-19 (with potential for a two- to four-fold increase) and dedicated bedside critical care physicians to serve these patients.

In this article, we review how HMH implemented its telecritical care program by focusing on the broader innovative application of this virtual technology to find patient-centric solutions while protecting staff and patient families during an extraordinary pandemic situation. The documented work has been identified as exempt by the institutional review board at HMH.

### Background

Telecritical care has revolutionized the delivery of care by enabling remote monitoring and treatment of ICU patients. Since their first pilot implementation in 1997 [2], telecritical care platforms, initially known and trademarked as electronic intensive care units (eICUs), have extended access to resources (eg, critical care physicians, specialized consults, and nurses), provided a wide range of decision-support tools, and enabled monitoring and analysis of a large amount of physiological data [3]. Recent studies show large variability in the mortality impacts of telecritical care platforms; for instance, in a 2016 national effectiveness study by Kahn et al [4], the positive impacts on the length of stay, cost, and quality of care contributed to increased popularity and adoption rates of telecritical care (see also [5,6]). A 2017 survey of 722 hospitals worldwide (672 in the United States) showed that 35% had formal telecritical care programs [6].

### The HMH vICU

**Figure 2:**
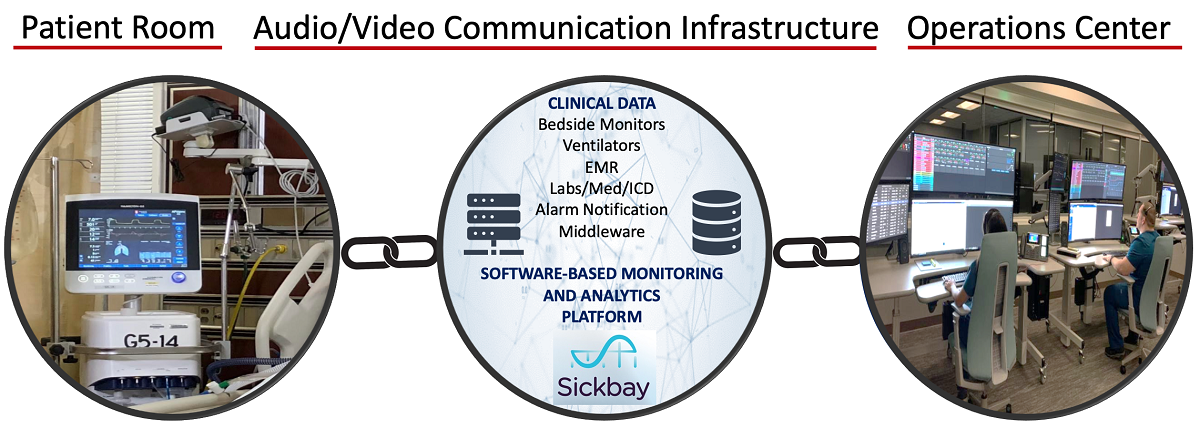
The three main components of the virtual intensive care unit system. AV: audiovisual; EMR: electronic medical record; ICD: Implantable Cardioverter-Defibrilators.

Before the onset of the COVID-19 pandemic, HMH launched its innovative telecritical care program, branded as the Virtual Intensive Care Unit (vICU), to augment the critical care services being provided in its ICUs. The HMH vICU has three main components: the operations center, the patient room, and the audiovisual (AV) communication infrastructure linking the first two components (Figure 2). The operations center, located at HMH’s main campus at the TMC, provides a central command capability where medical doctors and registered nurses, referred to as Virtual MDs (vMDs) and Virtual RNs (vRNs), connect and monitor the status of patients using the AV equipment installed in the patients’ rooms. The AV communication infrastructure includes a single camera in the patient rooms with 360-degree pan, tilt, and zoom capability that enables the vMDs and vRNs to examine the patient; they can also focus on intravenous fluids, drip rates, monitors, and ventilator settings, and, of course, they can communicate with the patients, their visiting families, and the bedside providers.

**Figure 3:**
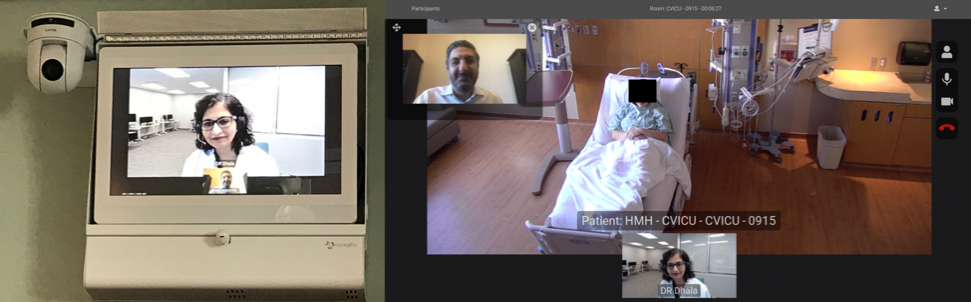
Virtual intensive care unit audio and video equipment installed in an intensive care unit room (left) and caller view (right). For the purposes of illustration, a member of the hospital staff played the role of the patient and one of the authors played the role of the caller.

Our operations center uses a US Food and Drug Administration–cleared software-based monitoring and analytics platform called Sickbay (Medical Informatics Corp). The physiological data from several bedside monitors and devices, including ventilators and hemodynamic monitors, and the static data from the electronic medical record (EMR) interface with this platform. Novel algorithms transform these big data into actionable information in the form of risk scores, which feed into clinical decision support systems. The operations center is also where virtual AV connections are established with the patients’ rooms. For emergent or urgent calls, the bedside teams can access the vICU by pressing a virtual alert button in each room to engage a vMD or vRN from the operations center. The vMDs and the vRNs can connect by camera within seconds to respond. In addition, the operations center can connect a consultant or an expert from any remote location through an application called Consultant Bridge. The operations center staff can send an SMS text message or email a link to the consultant to “bridge” them into the patient’s room. Consultant Bridge enables the vMD, the consultant, and the bedside team to have a three-way video call using the cameras and monitors already installed in the ICU patient rooms (Figure 3).

Initially, the vICU staff, consisting of one vMD and two vRNs, provided nocturnal coverage for HMH’s 36-bed neurology ICU (Neuro-ICU). To ensure a smooth transition and to minimize any disruptions to existing processes, the implementation plan called for a stepwise rollout of vICU to the other three units: the medical ICU (MICU), cardiovascular ICU (CVICU), and cardiac ICU (CICU). The vICU process required that all the staff’s expectations be clearly defined and workflows integrated appropriately while nurturing a robust and productive relationship with the bedside teams. The vICU and the ICU teams initiated a collaborative effort to design the essential workflows almost four months before the actual launch of the vICU.

## Methods

### ICU Expansions in Response to the COVID-19 Surge

The expected surge of the COVID-19 pandemic necessitated a sudden change in the rollout plan for the vICU. To fully prepare for the surge, HMH forecasted the number of beds that might be needed during the peak of the outbreak. At the time, the vICU had already started providing coverage to the Neuro-ICU during the night shift. The 24-bed MICU was the next unit scheduled to go live with vICU coverage. Instead, on March 12, 2020, the MICU became the first dedicated COVID-19 ICU at HMH. The novelty of SARS-CoV-2, combined with the complexity of treating COVID-19 patients, forced HMH to redirect all its critical care intensivists to the COVID-19 units, providing 24-hour bedside coverage.

### Staffing Changes in Response to the COVID-19 Surge

By March 26, 2020, the first COVID-19 ICU had almost reached capacity; this required the establishment of a second dedicated COVID-19 ICU, which was created by converting the 36-bed Neuro-ICU. By April 8, 2020, the Neuro-ICU had also reached full capacity, and a third COVID-19 ICU was set up by converting a 19-bed step down unit. For all the COVID-19 ICUs, the staffing ratio of intensivists to patients was 1:8 to 1:12 during the day and 1:12 to 1:18 during the night. Because the hospital had redirected the entire critical care physician workforce to the COVID-19 ICUs, the non–COVID-19 ICUs were managed by cardiovascular surgeons, cardiologists, neurosurgeons, and acute care surgeons.

To fill out the newly depleted critical care expertise in the non–COVID-19 ICUs, HMH expanded the night vICU program to provide urgent, emergent, and code blue support for all ICUs, including the COVID-19 ICUs. The bedside teams in the non–COVID-19 ICUs included advanced practice providers (APPs) who reached out to the vMDs for critical care support and admitted medicine overflow patients with the vMDs at night. HMH’s next step in the surge planning was to open the vICU in the daytime, providing critical care support from vMDs and vRNs during the day for the non–COVID-19 ICUs.

In the first week of April 2020, HMH began surveillance testing of its medical staff, and many frontline employees tested positive for COVID-19. All the COVID-19–positive workers, although asymptomatic, were removed from their clinical duties in the ICUs, and the resulting staff shortages expedited the integration of the vICU with the rest of the ICUs. Before the staff shortages, the vICU staff were only providing support for emergent, urgent, and code blue situations. However, the expanded vICU staff began to cover admissions and triage during the night for more comprehensive integration.

## Results

### ICU Expansions in Response to the COVID-19 Surge

It is well documented that family engagement has an enormous positive impact on ICU patients by decreasing anxiety, confusion, agitation, and delirium [7-9]. Evidence suggests that separating families from the patients can adversely impact the patient’s feelings of security and of ultimate outcome [10].

During the COVID-19 pandemic, nearly all hospitals disallowed visitors for adult inpatients, including all COVID-19 and non–COVID-19 ICU patients. While video chat technology (eg, FaceTime and Skype) is commonly available among patients and their families, in an ICU setting with a highly infectious, critically ill cohort of patients who may be sedated and frequently intubated, the use of this common technology was not feasible because it would require staff to bring in and position the equipment (eg, smartphone or tablet) while using personal protective equipment (PPE).

However, the vICU infrastructure provided a readily available and much more accessible means of connecting the patients with COVID-19 with their families. HMH began to offer this technology to the families for emotional support and improved patient care. Two vRNs (in the operation center) were tasked with reaching out to the bedside teams and collaborating with the bedside nurses, physicians, and unit managers to gain access to the patients’ families. Family members received links on their smartphones that instantly connected them with their loved one’s ICU room using the Consultant Bridge feature. The vICU patients logged approximately 20 to 40 calls per day using the Consultant Bridge during this period. Because restrictions on visitors also applied to non–COVID-19 patients and their families, the Consultant Bridge was used for all ICU patients. The results of a short postcall quality assessment survey showed overwhelming satisfaction with the access to the patient using vICU technology.

### Palliative Care for ICU Patients

In standard palliative care situations, family members are involved in the decision-making process at the bedside. However, in a pandemic situation, when the family members cannot be near their terminally ill loved ones, vICU technology may provide a vehicle to perform the critical steps with the full participation of the palliative care team and the family members. Similar to regular visitations, we used the Consultant Bridge feature of vICU to enable palliative care.

### Staffing Changes in Response to the COVID-19 Surge

**Figure 4:**
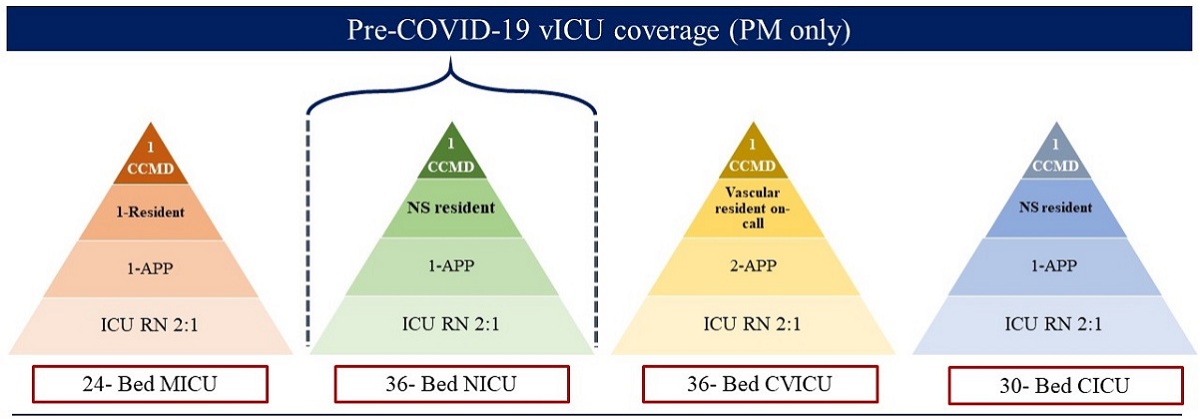
Houston Methodist Hospital tiered staffing model for critical care services supported by the virtual intensive care unit prior to the COVID-19 pandemic. APP: advanced practice provider; CCMD: critical care intensivist; CICU; cardiac intensive care unit; CVICU: cardiovascular intensive care unit; ICU: intensive care unit; MICU: medical intensive care unit; NS: neurosurgery; RN: registered nurse; vICU: virtual intensive care unit.

The demands of the COVID-19 pandemic resulted in the establishment of a tiered staffing model, with vICU providing essential support to all the ICUs. This model bears a strong resemblance to the tiered staffing model described by the Society of Critical Care Medicine for surge planning [11]. Prior to the COVID-19 pandemic, HMH had commenced its vICU program, providing nocturnal coverage for the Neuro-ICU combined with full workflow implementation. During this period, HMH used four in-house intensivists and the following staffing ratios: 2:1 for ICU RNs; 1 APP for 24 beds; 1 resident on call in the night; and 1 critical care bedside physician per unit (4 in total) (Figure 4).

There are many consultants whose presence at the bedside is not absolutely necessary during this pandemic despite their useful contributions to patient care plans. For these cases, vICU was able to bridge these consultants and their expertise to patient rooms without exposing the consultants to a highly infectious disease or requiring the expenditure of scarce PPE. The superior quality of the real-time video feed coupled with advanced zoom capability greatly enhanced the quality of these remote consults. For example, when a patient who tested positive for COVID-19 developed a maculopapular rash, the bedside team was able to perform a skin biopsy with the guidance of a dermatologist connected through the Consultant Bridge. The high quality of video transmission enabled the dermatologist to clearly see the skin lesion and direct the procedure.

**Figure 5:**
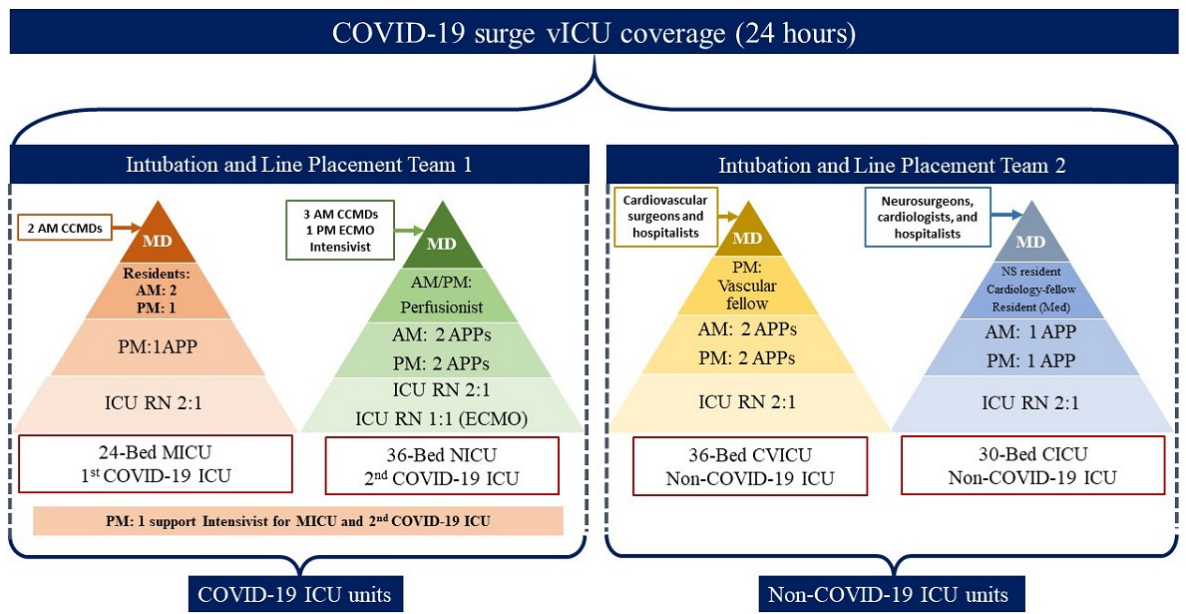
Houston Methodist Hospital tiered staffing model for critical care services supported by a virtual intensive care unit during the COVID-19 pandemic. APP: advanced practice provider; CCMD: critical care intensivist; CICU: cardiac intensive care unit; ECMO: extracorporeal membrane oxygenation; ICU: intensive care unit: MD: medical doctor; Med: Medical; MICU: medical intensive care unit; NICU: neurology intensive care unit; RN: registered nurse: vICU: virtual intensive care unit.

To adapt to the needs of patients with COVID-19, HMH expanded its vICU coverage from one unit to all units. HMH assigned all the intensivists to serve patients with COVID-19, with one support intensivist covering both COVID-19 ICUs at night. For the non–COVID-19 ICUs, coverage was provided by non–critical care attending physicians, mostly specialty surgeons, supported by vMDs and vRNs (Figure 5).

## Discussion

### Principal Findings

The experiences of HMH clinicians show that telecritical care platforms can be an essential part of the critical care services toolkit. While the benefits of having bedside intensivists have long been well known, telecritical care experts can support and augment the work of bedside teams treating critically ill patients, thereby expanding the scope and boundaries of traditional ICUs. Under normal conditions, telecritical care services may help reduce staff burnout in ICUs, lower ICU mortality rates, reduce length of stay in the hospital or ICU, and promote greater adherence to best practices.

During the COVID-19 pandemic, the challenges of treating patients with a highly infectious disease brought several novel applications of HMH’s vICU into sharp relief with positive patient outcomes. While HMH’s response to the COVID-19 pandemic quickly paved the way for the full integration of the vICU with all its ICUs, more work is needed to improve preparedness for and resilience in future pandemics. In particular, frequent drills [12] and simulation training [13] have shown promise in preparing ICU staff for rare but high-consequence pandemic events. These efforts can focus on identifying necessary adjustments to staffing, layout, processes, and new use cases for vICU technology. Participatory ergonomics methods and models such as technology acceptance have shown promise in eliciting ICU staff acceptance and intention to use these technological innovations [14].

One of the most important serendipitous benefits came from connecting patients to their family members through the use of the vICU platform via the Consultant Bridge application. Patients with COVID-19, who were isolated and separated from their families due to their highly infectious disease, were able to virtually see and hear their loved ones, bolstering the mental and emotional status of those patients. Many families expressed gratitude for this ability to see and speak with their loved ones. We observed tremendous potential in the use of this technology, and we believe that even non-ICU units may benefit from some form of telepresence technology to facilitate family engagement. While the Health Insurance Portability and Accountability Act (HIPAA) and consent regulations have been relaxed in light of the current pandemic, our vICU platform, including the Consultant Bridge, has been tailored to be HIPAA-compliant. In addition, the bedside staff ensure that the patient is “camera-ready” before any camera in the room is allowed to go live. Despite challenges related to privacy and security, a recent attitude shift toward viewing family members as partners in shared decision-making [15] has set the stage for a potential “open” vICU.

One of the significant contributors to burnout among HMH ICU providers is the anxiety associated with PPE shortages and exposure [16]. The use of the vICU provided a unique level of protection to the medical staff assigned to COVID-19 units. The ICU staff were given local access to patient rooms by vICU-enabled laptops that allowed the bedside physicians and nurses to connect to the patient’s room by camera without having to don and doff protective gear each time when checking on a patient. Such remote access reduced the usage of PPEs, which were already in short supply. The vICU’s Consultant Bridge also allowed specialty consultants, including extracorporeal membrane oxygenation specialists, cardiologists, and endocrinologists, to examine patients with COVID-19 virtually.

### Installation

A financial cost-benefit analysis of the vICU installation is beyond the scope of this paper. However, it should be noted that physical installation of the cameras, the alert buttons, and the rest of the communication infrastructure requires careful planning, including moving current patients while the rooms are being retrofitted with the equipment. Proper planning and advanced notification of the clinical teams are essential to remove any risk of disruption to patient care during the installation process.

### Scalability

With the ongoing resurgence of COVID-19 cases, HMH has been able to scale its ICU bed capacity by leveraging its vICU resources. The hospital converted previously mothballed ICU beds or intermediate care units into functioning ICU COVID-19–specific units by rolling out a mobile vICU “cart” that contains the camera and other communication hardware. These carts can be moved into any room or near any bed, providing instantaneous vICU connectivity to the vICU operation center and staff. While they are not a perfect substitute for a fully fitted vICU patient room, these mobile vICU carts provide great flexibility in deploying the vICU resources to newly converted ICU units or emergency rooms where critically ill patients may be waiting for an ICU bed.

More importantly, the structure of the vICU platform enables a high degree of scalability, as each vMD can provide coverage for up to 200 ICU patients. The patient-to-vMD ratio may vary among hospitals and health care systems. Finally, the vICU platform also enables our hospital system to use critical care MDs or APPs who may be high-risk individuals or may have tested positive for COVID-19 without requiring them to enter an ICU or even a hospital.

### Effectiveness of Reducing Infection Rate

The effectiveness of a vICU program must be measured in a broader strategic framework that can quantify how the vICU platform has expanded the availability of ICU beds and leveraged the staffing of critical care experts. At HMH, the vICU has allowed the hospital to significantly expand its number of beds without compromising the quality of patient care.

While we did not include empirical data regarding infection control in this study, the inherent structure of delivering critical care remotely protects the vICU providers as well as the consultants and family members, who do not come in contact with the patient with an infectious disease.

### Limitations

During the surge period, all the critical care physicians were conscripted to work in the COVID-19 ICUs, leaving the non–COVID-19 patients to be managed by non–critical care physicians and surgeons with support and oversight by critical care specialists in the vICU. While this adaptive behavior was a good indicator of resilience, the development of protocols informed by simulated and proactive efforts may provide more explicit guidelines for future staffing adjustments. In addition, while the tiered staffing model documented here shows promise for future pandemic surge planning, more work is warranted to improve this model and to develop generalizable guidelines for optimal and flexible allocation of resources during a pandemic.

### Conclusions

Telecritical care has been established as an advantageous mechanism for the delivery of critical care expertise. The current COVID-19 pandemic has brought multiple new useful applications to light that could be transformative in how telecritical care is perceived and deployed in the future, especially during highly infectious disease outbreaks. However, further research is required to examine the impact of innovative applications of telecritical care in the treatment of critically ill patients.

## Abbreviations

APP: advanced practice provider
AV: audiovisual
CICU: cardiac intensive care unit
CVICU: cardiovascular intensive care unit
eICU: electronic intensive care unit
EMR: electronic medical record
HIPAA: Health Insurance Portability and Accountability Act
HMH: Houston Methodist Hospital
ICU: intensive care unit
MICU: medical intensive care unit
Neuro-ICU: neurology intensive care unit
PPE: personal protective equipment
TMC: Texas Medical Center
vICU: virtual intensive care unit
vMD: virtual medical doctor
vRN: virtual registered nurse

## References

[1] Santangelo G (2020). New NIH resource to analyze COVID-19 literature: The COVID-19 portfolio tool. National Institutes of Health Extramural Nexus.

[2] Rosenfeld BA, Dorman T, Breslow MJ, Pronovost P, Jenckes M, Zhang N, Anderson G, Rubin H (2000). Intensive care unit telemedicine: alternate paradigm for providing continuous intensivist care. Crit Care Med.

[3] Celi LA, Hassan E, Marquardt C, Breslow M, Rosenfeld B (2001). The eICU: it's not just telemedicine. Crit Care Med.

[4] Kahn JM, Le TQ, Barnato AE, Hravnak M, Kuza CC, Pike F, Angus DC (2016). ICU Telemedicine and Critical Care Mortality: A National Effectiveness Study. Med Care.

[5] Udeh C, Udeh B, Rahman N, Canfield C, Campbell J, Hata JS (2018). Telemedicine/Virtual ICU: Where Are We and Where Are We Going?. Methodist Debakey Cardiovasc J.

[6] Subramanian S, Pamplin JC, Hravnak M, Hielsberg C, Riker R, Rincon F, Laudanski K, Adzhigirey LA, Moughrabieh MA, Winterbottom FA, Herasevich V (2020). Tele-Critical Care: An Update From the Society of Critical Care Medicine Tele-ICU Committee. Crit Care Med.

[7] Volland J, Fisher A, Drexler D (2015). Delirium and Dementia in the Intensive Care Unit: Increasing Awareness for Decreasing Risk, Improving Outcomes, and Family Engagement. Dimens Crit Care Nurs.

[8] Ely EW (2017). The ABCDEF Bundle: Science and Philosophy of How ICU Liberation Serves Patients and Families. Crit Care Med.

[9] Marra A, Ely EW, Pandharipande PP, Patel MB (2017). The ABCDEF Bundle in Critical Care. Crit Care Clin.

[10] Giannini A (2010). The "open" ICU: not just a question of time. Minerva Anestesiol.

[11] Halpern NA, Tan KS, Society of Critical Care Medicine Ventilator Taskforce (2020). US ICU resource availability for COVID-19. Version 2. Society of Critical Care Medicine.

[12] King MA, Niven AS, Beninati W, Fang R, Einav S, Rubinson L, Kissoon N, Devereaux AV, Christian MD, Grissom CK, Task Force for Mass Critical Care (2014). Evacuation of the ICU: care of the critically ill and injured during pandemics and disasters: CHEST consensus statement. Chest.

[13] Brazzi L, Lissoni A, Panigada M, Bottino N, Patroniti N, Pappalardo F, Gattinoni L (2012). Simulation-based training of extracorporeal membrane oxygenation during H1N1 influenza pandemic: the Italian experience. Simul Healthc.

[14] Kowitlawakul Y (2011). The technology acceptance model: predicting nurses' intention to use telemedicine technology (eICU). Comput Inform Nurs.

[15] Azoulay E, Chaize M, Kentish-Barnes N (2014). Involvement of ICU families in decisions: fine-tuning the partnership. Ann Intensive Care.

[16] Sasangohar F, Jones SL, Masud FN, Vahidy FS, Kash BA (2020). Provider Burnout and Fatigue During the COVID-19 Pandemic: Lessons Learned From a High-Volume Intensive Care Unit. Anesth Analg.

